# Changes in the state of matter of KCIO_4_ to improve thermal and combustion properties of Al/MoO_3_ nanothermite

**DOI:** 10.1186/s13065-024-01202-6

**Published:** 2024-05-09

**Authors:** Jialin Chen, Shutao Li, Mengnan Dai, Ming An, Rui Song, Yeqing Chen, Jiaxing Song, Quanwei Tian, Xiting Zhong, Qiushi Yan

**Affiliations:** 1 Institute of Defense Engineering, AMS, PLA, Beijing, China; 2 School of Physics, Southeast University, Nanjing, China; 3 Department of General Education, Army Engineering University of PLA, Nanjing, China; 4 Xi’an Rare Metal Materials Research Institute Co., Ltd, Xi’an, China; 5 Key Laboratory of Urban Security and Disaster Engineering under the Ministry of Education, Beijing University of Technology, Beijing, China

**Keywords:** Thermite, Constant volume combustion, Melt and decomposition, Thermodynamics analysis

## Abstract

To improve the thermal and combustion properties of nanothermites, a design theory of changing the state of matter and structural state of the reactants during reaction was proposed. The Al/MoO_3_/KClO_4_ (Kp) nanothermite was prepared and the Al/MoO_3_ nanothermite was used as a control. SEM and XRD were used to characterize the nanothermites; DSC was used to test thermal properties; and constant volume and open combustion tests were performed to examine their combustion performance. Phase and morphology characterization of the combustion products were performed to reveal the mechanism of the aluminothermic reaction. The results show that the Al/MoO_3_/Kp nanothermite exhibited excellent thermal properties, with a total heat release of 1976 J·g^− 1^, increasing by approximately 33% of 1486 J·g^− 1^ of the Al/MoO_3_ nanothermite, and activation energy of 269.66 kJ·mol^− 1^, which demonstrated higher stability than the Al/MoO_3_ nanothermite (205.64 kJ·mol^− 1^). During the combustion test, the peak pressure of the Al/MoO_3_/Kp nanothermite was 0.751 MPa, and the average pressure rise rate was 25.03 MPa·s^− 1^, much higher than 0.188 MPa and 6.27 MPa·s^− 1^ of the Al/MoO_3_ nanothermite. The combustion products of Al/MoO_3_ nanothermite were Al_2_O_3_, MoO, and Mo, indicating insufficient combustion and incomplete reaction, whereas, the combustion products of Al/MoO_3_/Kp nanothermite were Al_2_O_3_, MoO, and KCl, indicating complete reaction. Their “coral-like” morphology was the effect of reactants solidifying after melting during the combustion process. The characterization of reactants and pressure test during combustion reveals the three stages of aluminothermic reaction in thermites. The excellent thermal and combustion performance of Al/MoO_3_/Kp nanothermite is attributed to the melt and decomposition of Kp into O_2_ in the third stage. This study provides new ideas and guidance for the design of high-performance nanothermites.

## Introduction

Nanothermite, a nanoscale fuel and oxidizer particle mixture, has a shorter diffusion distance than conventional micron-sized or monomolecular energetic materials. Therefore, it has a very high energy release rate, which releases a large amount of heat, demonstrating good combustion and thermal properties [1–6].

Currently, there are extensive published findings on oxidizers such as Al/MoO_3_, Al/NiO, Al/SiO_2_, Al/WO_3_, Al/CuO thermites, etc [7–13]. . . Among numerous oxides, MoO_3_ has unique chemical and physical properties for applications in the fields of optics, electronics, catalysis, biology, energy systems [14–17], etc. More importantly, it holds an advantage in the application of thermites. Stoichiometric ratio calculation implies that the exothermic enthalpy of MoO_3_ is higher than most metal oxides. Research has shown that Al/MoO_3_ nanothermite has better ignition performance [14], therefore, as an oxide in thermites, more potentials of MoO_3_ remain to be investigated. Thanks to its high heat, high redox potential, and rapid reactivity [18, 19], nano Al has been used as the main constituent of solid rocket propellants and other propulsion systems [18]. However, Al nanoparticles are prone to agglomeration adhesion [20], which has a great impact on the process of aluminothermic reaction. The sintering products from local precombustion may block the combustion progress [21, 22], and hinder the self-propagating combustion of thermite, keeping heat from complete release.

Therefore, maintaining a good dispersion of nano Al or designing a novel structure with a large interface contact area can greatly improve the reactivity of fuel with the oxidant. To increase the contact area of reactants, various preparation methods and strategies have been investigated, such as sol-gel synthesis [23], vapor deposition [24], self-assembly [3], high-energy ball milling [25], etc. A variety of thermite structures have been prepared, including 3D-ordered array nanothermite film [26], core-shell structures [27], etc. Novel preparation methods and structures can effectively improve combustion characteristics and greatly increase safety yet with the disadvantages of cumbersome operation techniques and high preparation costs.

The foregoing studies aimed to increase interfacial reactions by modulating the initial structure of reactants, thereby improving the thermal and combustion properties. However, is it possible to use the structural changes in reaction to increase the contact area? For this purpose, a nanothermite that produces gas and undergoes changes in the state of matter during the reaction can be designed. The addition of gas-producing agents is an outstanding method. The agents generate gases during the reaction, thus increasing the pressure in the reaction system, prompting closer contact at the reaction interfaces and increasing the overall reactivity, e.g., nitrocellulose (NC) [28], copper oxide (CuO) [29], bismuth oxide (Bi_2_O_3_) [30], etc. In terms of the reactant states, most of the current thermites undergo a solid-solid reaction during the aluminothermic reaction [31, 32], whereas few studies have been reported on the changes in the state of matter during the main reaction.

Potassium perchlorate (KClO_4_, Kp) is an excellent additive that decomposes into O_2_ under high-temperature conditions, which has a melting temperature close to the reaction temperature of nanothermites. Therefore, it has been shown that as an excellent additive, it can substantially improve the combustion performance of thermites [33, 34]. However, until recently, there have been few studies on the mechanism of why it can promote the reaction of thermites, and the specific reaction path has not yet been revealed. Further systematic exploration is still needed.

In this study, the hydrothermal method was used to prepare nano-MoO_3_, and the ultrasonic dispersion method was used to prepare Al/MoO_3_/Kp nanothermite. The prepared nanothermite was tested for thermal and combustion properties, and thermodynamics analysis was performed to calculate activation energy. The combustion products were collected to investigate the reaction state of the aluminothermic reaction process and the reaction mechanism of Al/MoO_3_/Kp nanothermite. Moreover, Al/MoO_3_ nanothermite was prepared for comparison.

## Experiment

### Material preparation

(NH_4_)_6_Mo_7_O_24_·4H_2_O (99% purity) was provided by Shanghai Bide Pharmatech Ltd. KClO_4_ (Kp) and nano Al powder were provided by Aladdin Industries. Dimethylformamide (DMF, 99.8%), anhydrous ethanol (99.7%), deionized water, and HNO_3_ (concentration 68%) were provided by Sinopharm Chemical Reagent Co., Ltd. All reagents were used directly without any treatment.

### Preparation of nano-MoO_3_

**Fig. 1:**
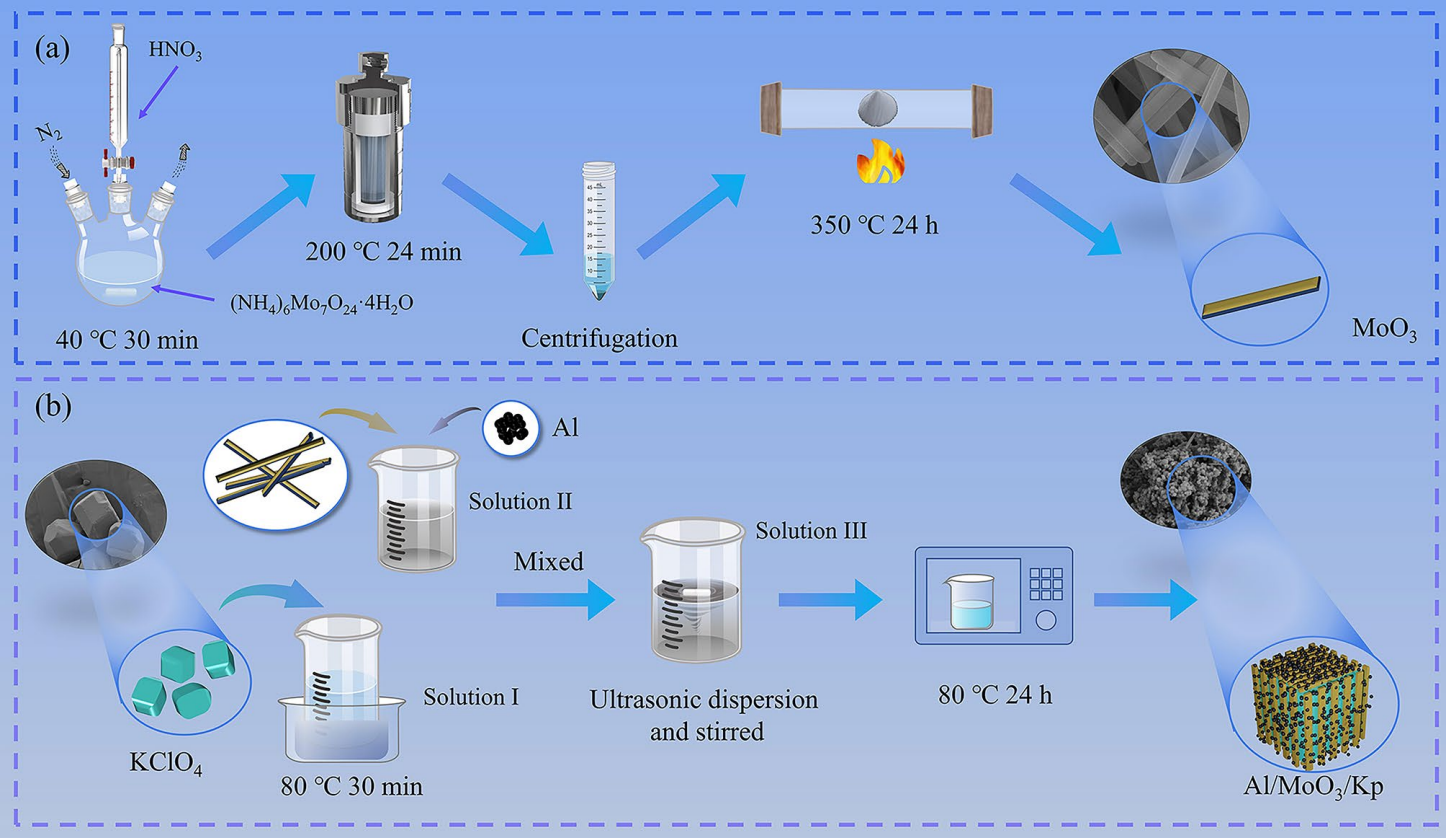
Preparation flow chart (**a**) nano-MoO_3_ preparation flow chart (**b**) nanothermite preparation flow chart

Nano-MoO_3_ was prepared using the hydrothermal method, and the preparation process is shown in Fig. 1(a). The early chemical reaction took place in an N_2_ atmosphere. To ensure the smooth progress of the reaction, a suitable installation was set up using vessels such as flasks and constant pressure funnels. First, an ammonium molybdate solution was prepared using 1.5 g (NH_4_)_6_Mo_7_O_24_·4H_2_O and 30 ml of deionized water, and magnetically stirred for 30 min at 40 ^o^C in a water bath. Afterwards, the diluted HNO_3_ solution was added drop by drop to the ammonium molybdate solution, and the chemical reaction was ensured to take place under the N_2_ atmosphere. With the addition of the acid solution, the color of the solution changed from colorless to milky white and then to clear colorless. After the reaction was completed, the solution was moved to a high-pressure reactor lined with polytetrafluoroethylene, and heated at 200 ^o^C for 24 h. Subsequently, the solution was washed three times with deionized water and centrifuged. The precipitates were dried and heated at 350 ^o^C for 24 h before being ground to obtain the nano-MoO_3_ sample. The main chemical reactions are shown in formula (1):

1 $${\text{M}}{{\text{o}}_{\text{7}}}{\text{O}}_{{{\text{24}}}}^{{{\text{6-}}}}{\text{+6}}{{\text{H}}^{\text{+}}} \to {\text{7Mo}}{{\text{O}}_{\text{3}}}{\text{+3}}{{\text{H}}_{\text{2}}}{\text{O}}$$

### Nanothermite preparation

Due to the existence of an oxide layer, the activity of the nano Al powder will not be 100% [35]. The activity of nano Al powder was measured before the preparation of nanothermite. To analyze the content of active Al, tests were conducted using a synchronous thermal analyzer (TG-DSC) at 30 ?∼ 1200 ^o^C, with a heating rate of 10 K·min^-1^ and an air flow rate of 50 ml·min^-1^ to ensure complete oxidation of the Al nanoparticles. The oxidation reaction of Al in air is shown in formula (2):

2 $$4{\text{Al}} + 3{{\text{O}}_2} = 2{\text{A}}{{\text{l}}_2}{{\text{O}}_3}$$

Figure 3(a) exhibits the TG-DSC profile of nano Al thermal analysis, which shows a significant increase in mass, reaching 158.22% of the original mass. The increase is attributed to the oxidation of active Al. Formula (3) can be used to calculate the content (*c*) of active Al:

3 $$c\left( \% \right)=\frac{{108}}{{96}}\Delta m\left( \% \right)$$

Where $\Delta m\left( \% \right)$ is the percentage of mass increase in the thermogravimetric analysis test. The measured content of active Al was 65.5% based on the test and calculation. The DSC profile of nano Al exhibits a distinct endothermic peak at 661^o^C, the melting endothermic peak of nano Al.

The mass of the oxidant remained unchanged. The ratio of oxidant to fuel in the thermite was allocated according to the zero oxygen balance in the chemical reaction, as shown in Table 1. Two nanothermites were prepared for comparison. In the Al/MoO_3_ nanothermite, the mass of nano-MoO_3_ was 120 mg. Based on the stoichiometric ratio calculation in chemical formula (4), the fuel Al required to reach zero oxygen balance for 120 mg of MoO_3_ was approximately 45 mg. However, the aluminum powder used was only 65.5% active, so the mass of the aluminum sample was 69 mg. In the Al/MoO_3_/Kp nanothermite, the masses of nano-MoO_3_ and Kp were 75 mg and 45 mg, respectively. Based on the calculations of the stoichiometric ratio and active Al percentage in chemical formulae (4) and (5), the required fuel Al was approximately 79 mg.

4 $$2{\text{Al}} + {\text{Mo}}{{\text{O}}_3} = {\text{A}}{{\text{l}}_2}{{\text{O}}_3} + {\text{Mo}}$$

5 $$3{\text{KCl}}{{\text{O}}_4} + 8{\text{Al}} = 4{\text{A}}{{\text{l}}_2}{{\text{O}}_3} + 3{\text{KCl}}$$

Nanothermite was prepared using the ultrasonic dispersion method and the preparation process is shown in Fig. 1(b). Kp was dissolved in 10 ml of DMF, heated in the water bath at 80 ^o^C for 30 min, and labeled as solution I, where Kp exhibited a bulk-like microscopic morphology as shown in Fig. 1(b). Nano-MoO_3_ and nano Al powder were dissolved in a mixture of 35 ml of DMF and 5 ml of anhydrous ethanol, magnetically stirred for 30 min, and labeled as solution II. Afterwards, the two solutions were mixed, magnetically stirred for another 30 min, and labeled as solution III. Solution III was ultrasonically dispersed for 40 min to obtain a homogeneously dispersed suspension. Finally, the suspension was dried at 80 ^o^C for 24 h to obtain the Al/MoO_3_/Kp nanothermite sample. Solution II was also ultrasonically dispersed for 40 min and dried to obtain an Al/MoO_3_ nanothermite sample as the control group.

**Table 1:** Nanothermite oxidant and fuel ratio table

Sample	MoO_3_/mg	KClO_4_/mg	Al/mg
Al/MoO_3_ nanothermite	120	0	69
Al/MoO_3_/Kp nanothermite	75	45	79

### Characterization testing and thermal analysis

The sample structures were detected with X-ray diffraction (XRD, Bruker D8 Discover, Germany) at an operating voltage of 30 kV and characterized using CuKα radiation (λ = 0.1542 nm) at a scan rate of 10°/min.

Scanning electron microscopy (SEM, JSM-7800 F, Japan) was used to observe the morphology of the samples. The device was used for imaging at a voltage of 5 kV and a magnification of 1 ?∼ 100,000 times.

Differential scanning calorimetry (DSC, NETZSCH STA 449F3, Germany) was used to measure the thermal properties of the samples. Thermal analysis of the thermites was conducted in an argon atmosphere, with a temperature range of 40℃?∼1000℃ and a heating rate of 25 K·min^− 1^. To ensure safety, the mass of the sample was 3.0 mg each time. Thermal analysis tests were performed at different heating rates of 10, 15, 20, and 25 K·min^− 1^ for thermal dynamics analysis for the thermites.

### Combustion test

**Fig. 2:**
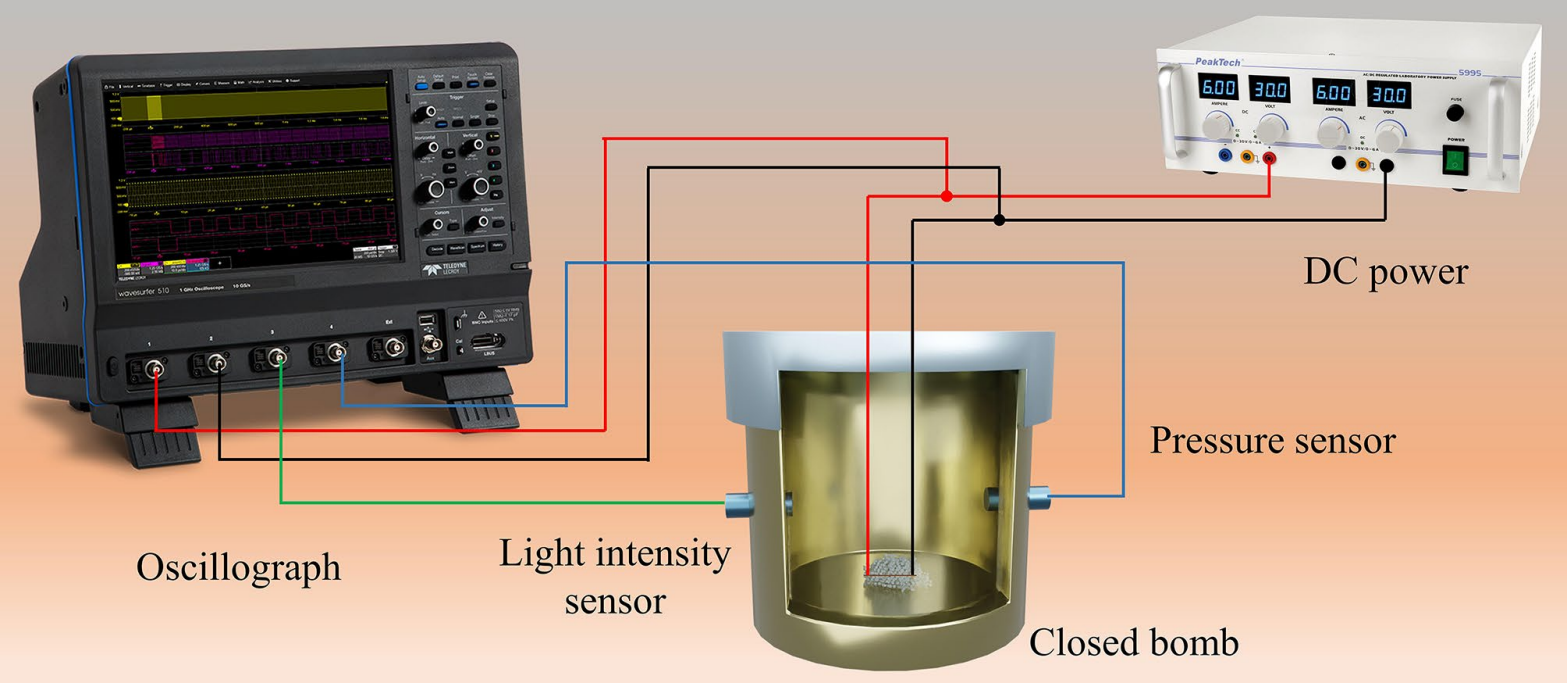
Constant volume combustion test

The constant volume combustion test is shown in Fig. 2. An adjustable DC power supply was used to quickly heat a nickel-chromium wire with a diameter of 0.1 mm to ignite the thermite sample, and the experimental voltage and current were recorded using an oscilloscope (Wavesurger 3054). The closed bomb was equipped with a pressure sensor and a light intensity sensor to measure the shock wave pressure and light intensity generated by sample combustion, which was recorded with an oscilloscope. The current transfer ratio of the current detector was 1 V/A, and the relationship between pressure and voltage was *P* = 5.63 V. To ensure safety, a small dose of thermite sample was used with a mass of 15 mg.

To observe the combustion state of thermites clearly, open combustion tests were also performed. In an open environment, the combustion process was recorded by a high-speed camera (FASTCAM SA-Z, Japan). The sampling frequency was 20,000 frames per second, the frame size was 768 × 768 pixels, and the aperture value was 3.2. The starting point was the time when the first firelight appeared and was denoted by 0.

## Results

### Characterization results

**Fig. 3:**
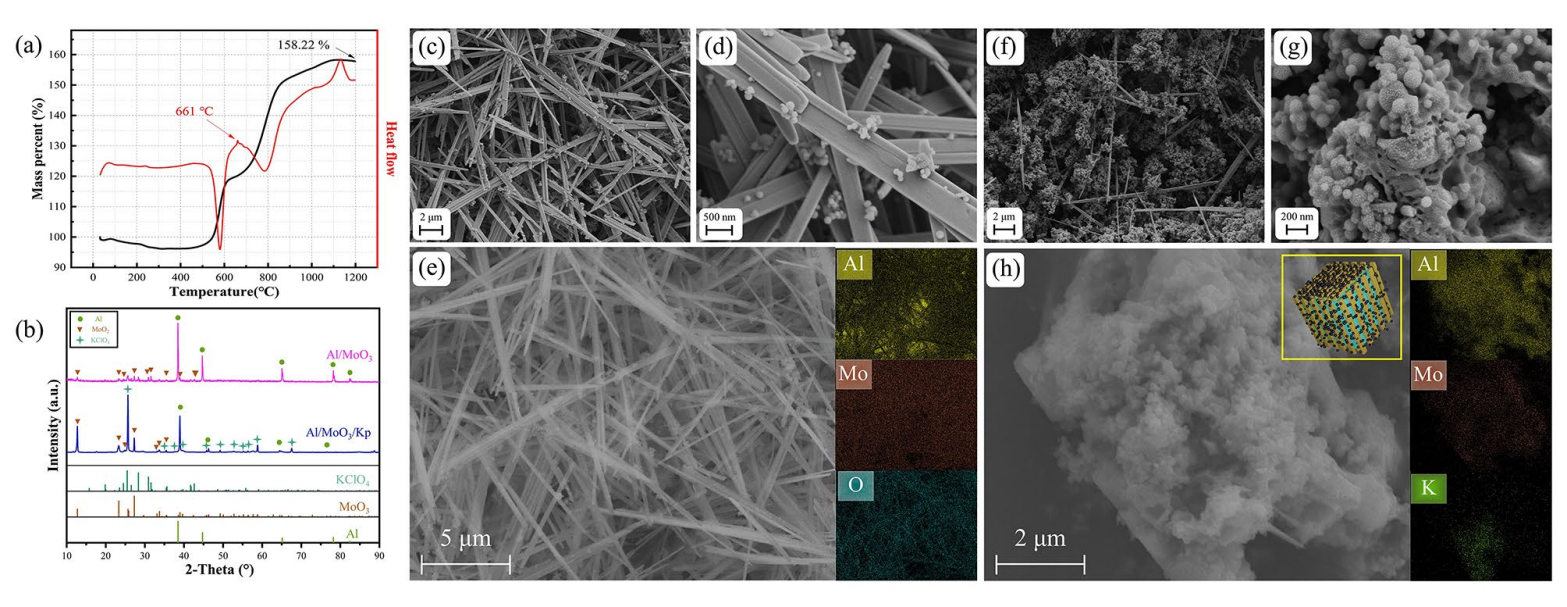
Characterization diagram of the nanothermites (**a**) TG-DSC curve of nano Al heated in air (**b**) XRD diagram of the nanothermites (**c**) SEM view of the Al/MoO_3_ nanothermite (**d**) Partial enlarged SEM view of the Al/MoO_3_ nanothermite (**e**) Mapping image of the Al/MoO_3_ nanothermite (**f**) SEM view of the Al/MoO_3_/Kp nanothermite (**g**) Partial enlarged SEM view of the Al/MoO_3_/Kp nanothermite (**h**) Mapping image of the Al/MoO_3_/Kp nanothermite

Figure 3(b) is the XRD diffraction pattern of the prepared nanothermite. According to the standard comparison PDF card, as can be seen from the Fig. 3(b), the peak position and peak intensity of the Al/MoO_3_ nanothermite diffraction peak match the standard phase cards MoO_3_ JCPDS No.76-1003 and Al JCPDS No. 04-0787. Similarly, the peak position and peak intensity of the Al/MoO_3_/Kp nanothermite diffraction peak are also consistent with the mentioned two standard cards. In addition, they are also consistent with the KClO_4_ JCPDS No. 70–0488 standard card, which proves that the prepared nanothermite has high purity and no obvious impurities.

The microstructure of the synthesized MoO_3_ is shown in the SEM image in Fig. 1(a), which exhibits a rod-shaped synthesized MoO_3_, with a diameter of 80 ?∼ 120 nm and a length of 15 ?∼ 20 μm. Its surface is smooth and growth is regular. The microstructures of the nanothermite samples were observed using SEM, and the results are shown in Fig. 3(c)?∼(h). Figure 3(c)?∼(e) are the SEM and Mapping images of the Al/MoO_3_ nanothermite, from which evenly distributed Al nanoparticles on the rod-shaped MoO_3_ can be observed. However, there is also agglomeration of Al nanoparticles. As is well known, aluminum aggregation is inevitable, which has been reported in previous studies [36, 37]. It can be seen from the Mapping in Fig. 3(e) that Al and MoO_3_ are evenly dispersed, indicating good dispersibility. In the distribution of O element, the stripes of the rod-shaped MoO_3_ can be observed, and Al is distributed along the MoO_3_ stripes, indicating that the Al/MoO_3_ nanothermite is uniformly mixed.

Figure 3(f)?∼(h) are the SEM and Mapping images of the Al/MoO_3_/Kp nanothermite. The synthesized nanothermite appears to have a coating-doping shape with KClO_4_ as its core and Al and MoO_3_ as its shell. Figure 3(f)?∼(g) show that Al particles and rod-shaped MoO_3_ are interlaced and adhered to each other in a doped arrangement. The dense Al nanoparticles and interlaced MoO_3_ have wrapped the bulk KClO_4_. The coating structure can be further demonstrated by Fig. 3(h), where the element distribution indicates that Al is widely distributed in the periphery of the wrapped area, followed by the Mo element in the middle layer, which represents the nano-MoO_3_. The K element was detected at the core, indicating a blocky KClO_4_ core layer. They form a coating structure as shown in the diagram of the upper right corner of Fig. 3(h).

### Thermal analysis results

**Fig. 4:**
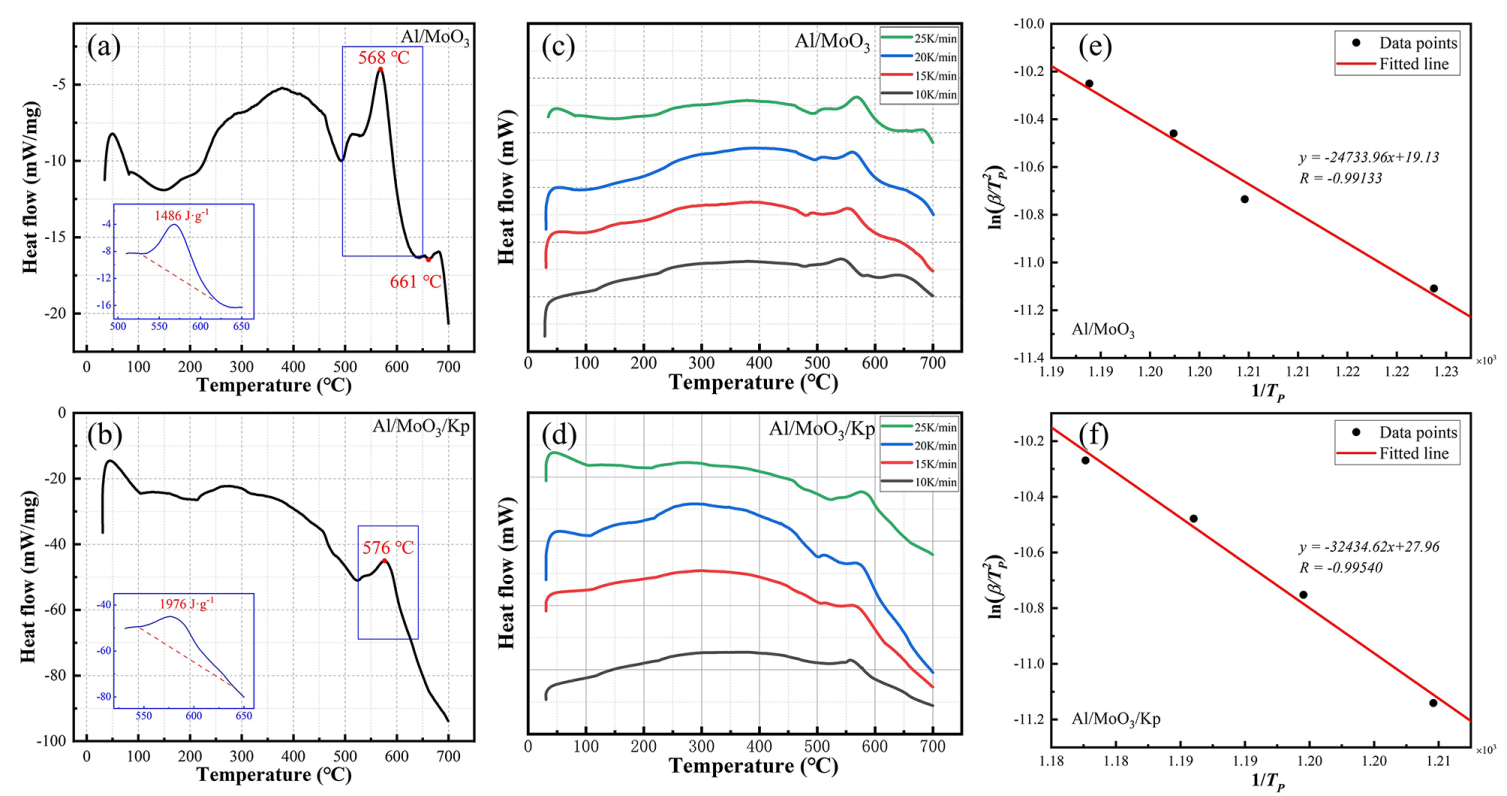
DSC curves of the nanothermites. DSC curves of the nanothermites at a heating rate of 25 K·min^− 1^ (**a**) Al/MoO_3_, (**b**) Al/MoO_3_/Kp; DSC curves of the nanothermites at different heating rates (**c**) Al/MoO_3_, (**d**) Al/MoO_3_/Kp; Activation energy fitting curves of the nanothermites (**e**) Al/MoO_3_, (**f**) Al/MoO_3_/Kp

Figure 4(a) and (b) exhibit the DSC curves of Al/MoO_3_ and Al/MoO_3_/Kp nanothermites at a heating rate of 25 K·min^− 1^, respectively. Over the 200 ?∼ 300^o^C range, the DSC curves both exhibit small endothermic peaks, which is possibly due to the evaporation of adsorbed water, structured water, solvents, or impurities on the surface of the thermite during its preparation [38]. Two main exothermic peaks both appeared at approximately 500^o^C, which were generated from the aluminothermic reaction.

The exothermic peak of Al/MoO_3_ nanothermite peaked at 568^o^C, as shown at the bottom-left of Fig. 4(a) in an enlarged picture, with a total exothermic heat of 1486 J·g^− 1^, whereas the exothermic peak of Al/MoO_3_/Kp nanothermite peaked at 576^o^C, with a total heat release of 1976 J·g^− 1^, which was approximately 33% higher than the Al/MoO_3_ nanothermite, demonstrating good thermal performance. In published studies [39], the Al/Kp nanothermite, when tested using DSC, exhibited an exothermic heat release of 245.6 J·g^− 1^, which is significantly lower than that observed for the Al/MoO_3_ nanothermites and the Al/MoO_3_/Kp nanothermites. This comparison unequivocally demonstrates that the notable improvement in the performance of the Al/MoO_3_/Kp nanothermite is primarily attributed to the catalytic promotional effect of Kp on the reaction mechanism of Al/MoO_3_, rather than merely being a simple additive effect of the two reactions. Their main heat release details are listed in Table 2. It is noteworthy that there is a small endothermic peak at 661^o^C in the DSC curve of the Al/MoO_3_ nanothermite. According to the DSC curve in Fig. 3(a), it is the melting endothermic peak of nano Al, demonstrating residual nano Al in the Al/MoO_3_ nanothermite and incomplete reaction. In contrast, there is no melting endothermic peak of Al in the curve of Al/MoO_3_/Kp nanothermite, wherein the nano Al had fully reacted.

Further, to investigate the thermal kinetics of the nanothermites, their activation energy (Ea) can be calculated according to the DSC curves of multiple heating rates in Fig. 4(c)?∼(d).

**Table 2:** The main details of DSC of nanothermite

Sample	T_onset_/^o^C	T_peak_/^o^C	H_exo_/J·g^− 1^
Al/MoO_3_ nanothermite	539	568	1486
Al/MoO_3_/Kp nanothermite	535	576	1976

The Kissinger [40] method was chosen to calculate the *Ea* of the thermites, as shown in Formula (6).

6 $$\ln \left( {\frac{\beta }{{T_{P}^{2}}}} \right)= - \frac{{{E_a}}}{{R{T_P}}}+\ln \frac{{AR}}{{{E_a}}}$$

Where *A* is the prefactor (s^− 1^), *R* is the universal gas constant (8.314 J·mol^− 1^·K^− 1^), *T*_*P*_ is the DSC peak temperature (K), *β* is the linear heating rate (K·min^− 1^). Assuming the conversion rates of the samples at the peak temperature are the same, the relationship between *ln (β/T*_*P*_^*2*^*)* and *1/T* is a straight line, the slope of which can be converted into an *Ea* value, and the intercept can be converted into an *A* value.

Figure 4(c)?∼(d) exhibit consistent curve trends under different heating rates, indicating good repeatability of the samples of thermal performance. The exothermic peak details of the Al/MoO_3_ and Al/MoO_3_/Kp nanothermites are listed in Table 3.

According to the details in Table 3, the experimental points of Al/MoO_3_ and Al/MoO_3_/Kp nanothermites were linearly fitted to obtain the slope equation, and then *Ea*. Figure 4(e) is the fitting result of Al/MoO_3_ nanothermite. The straight line equation is *y=-24733.96x + 19.13*, and the calculated Ea is 205.64 kJ·mol^− 1^. Figure 4(f) is the fitting result of Al/MoO_3_/Kp nanothermite. The straight line equation *y=-32434.62x + 27.96*, and the calculated *Ea* is 269.66 kJ·mol^− 1^. Activation energy is the energy required for a substance to change from a normal state to an active state that is prone to chemical reactions. The activation energy of Al/MoO_3_/Kp nanothermite is higher than Al/MoO_3_ by approximately 64.02 kJ·mol^− 1^, demonstrating better stability of the Al/MoO_3_/Kp nanothermite.

**Table 3:** The main details of DSC curves under different heating rates

Sample	Heating rate/K·min^− 1^	T_peak_/^o^C	T_peak_/K	E_a_/kJ·mol^− 1^
Al/MoO_3_ nanothermite	10	544	817	205.64
	15	557	830	
	20	562	835	
	25	568	841	
Al/MoO_3_/Kp nanothermite	10	557	830	269.66
	15	564	837	
	20	570	843	
	25	576	849	

### Combustion performance

**Fig. 5:**
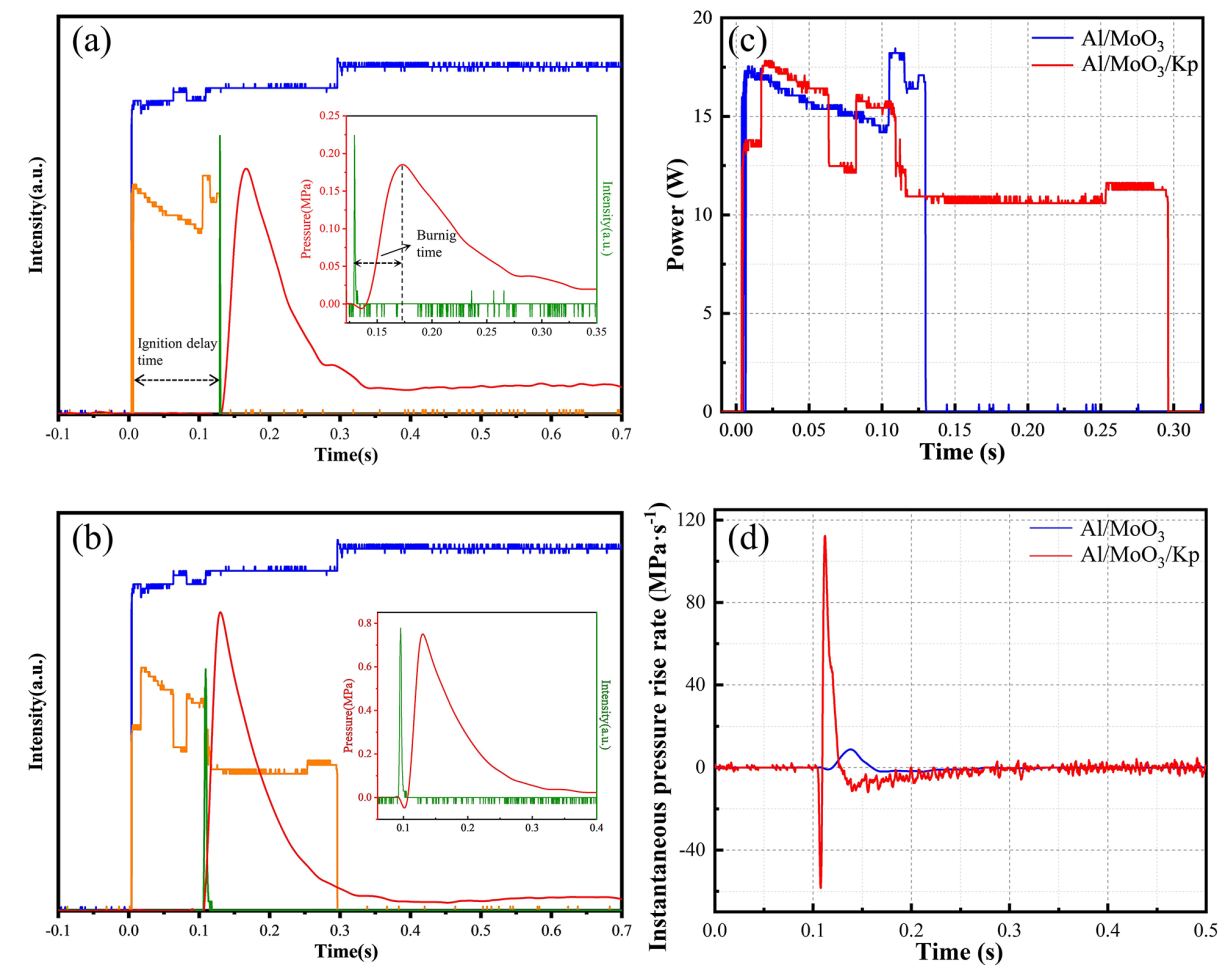
Curves of the constant volume combustion tests. Combustion waveform curves: (**a**) Al/MoO_3_, (**b**) Al/MoO_3_/Kp; (**c**) Power-time characteristic curves of the nanothermites in the ignition delay stage (d) Instantaneous pressure rise rate curves of the nanothermites

In the constant volume combustion experimental apparatus shown in Fig. 2, the oscilloscope could measure the waveform curves of the voltage (*U*) and current (*I*) flowing through the nickel-chromium wire, as well as that of the pressure (*P*) and light signal (*L*) of the nanothermite combustion. The recorded constant volume combustion test waveform curves of the Al/MoO and Al/MoO_3_/Kp nanothermites are shown in Fig. 5(a)?∼(b).

The time between the switch closure of the constant volume combustion apparatus and the ignition of the thermite samples is defined as the ignition delay time, as shown in Fig. 5(a). The energy consumed during this period is considered to be entirely used for stimulated ignition and is defined as ignition energy. The product of voltage and current is defined as ignition power, i.e., *P*_*ower*_*=UI*. The work done during the delay time is ignition energy, i.e., *W=∫UIdt*. The time from the generation of the light signal to the peak pressure is defined as the combustion time, as shown in Fig. 5(a). Since the pressure increased during the combustion of the thermite, when the pressure decreased, the thermite burned out. The ratio of the peak pressure to pressure rise time is defined as the average pressure rise rate. The constant volume combustion index is listed in Table 4.

The ignition performance of nanothermites reflects the degree of difficulty for them to be ignited under the action of external energy and is commonly measured by ignition temperature, ignition delay time, and ignition energy [41]. Figure 5(c) exhibits the power-time characteristic curves of the Al/MoO_3_ and Al/MoO_3_/Kp nanothermites in their ignition delay stage. Their instantaneous pressure rise rate curves are shown in Fig. 5(d), which are obtained by differentiating their pressure curves. After the ignition and delay time, the ignition combustion of the nanothermite samples was stimulated. The pressure in the bomb increased rapidly. The delay time of the Al/MoO_3_ nanothermite was 0.13 s, ignition energy was 2.004 J, pressure peak was 0.188 MPa, combustion time was 0.03 s, and its average pressure rise rate was 6.27 MPa·s^− 1^. The delay time of the Al/MoO_3_/Kp nanothermite was 0.10 s, ignition energy was 1.505 J, pressure peak was 0.751 MPa, combustion time was 0.03 s, and average pressure rise rate was 25.03 MPa·s^− 1^. The comparison reveals that the peak pressure and average pressure rise rate of the Al/MoO_3_/Kp nanothermite are much higher than those of the Al/MoO_3_ nanothermite, generating a large amount of gas during the reaction process, which is conducive to a faster reaction. In comparison, the Al/MoO_3_/Kp nanothermite has better combustion performance than the Al/MoO_3_ nanothermite. In studies detailing constant-volume combustion experiments with Al/Kp [42], the peak pressure recorded was approximately 0.214 MPa. Adding together the peak pressures for both Al/MoO_3_ nanothermite and Al/Kp does not reach the peak pressure observed with the Al/MoO_3_/Kp nanothermite. This evidence further highlights that the significant enhancement in the performance of the Al/MoO_3_/Kp nanothermite is not merely the cumulative effect of two separate reactions. Instead, the enhancement is attributed to the catalytic promotion by Kp on the Al/MoO_3_ reaction mechanism.

It is important to highlight that experimental data reveal the ignition energy needed for the Al/MoO_3_/Kp nanothermite is lower compared to that of the Al/MoO_3_ nanothermite. This finding may initially appear contradictory to the previously calculated activation energy, yet there is no conflict. Activation energy is the minimum energy threshold required to initiate a chemical reaction. It is a kinetic parameter that describes the energy barrier that molecules must overcome to transform from reactants to products during a chemical reaction. Ignition energy, on the other hand, typically refers to the energy that a molecule must absorb to reach an excited state from which a reaction can proceed. Typically, there is a relationship between excitation energy and activation energy, yet they operate on different orders of magnitude. Ignition energy has macroscopic characteristics, and its measured outcomes are influenced not only by activation energy but also by changes in external environmental factors. It should be noted that the three experiments were conducted under distinct conditions. The DSC analysis took place in an environment where argon gas was continuously introduced. For the open combustion experiment, there was air circulation. Both of these experiments were affected by the gas flow rate. In contrast, the constant-volume combustion test was executed within a sealed bomb calorimeter, with static internal gases. These conditions may lead to variations in ignition energy, with the primary influences comprising: (1) Heat accumulation: Within a closed system, the challenge of dissipating heat to the environment can result in higher temperatures in the reaction zone. This temperature rise assists in reaching the critical state necessary to overcome the activation energy more rapidly, thereby enabling the reaction without the need for extra ignition energy. (2) Pressure effect: Under constant-volume sealed conditions, gases produced during the reaction increase the internal pressure of the system. This increased pressure could impact the chemical equilibrium, especially in reactions involving gaseous products. (3) Physical state transitions of substances: In conditions of high temperature and/or pressure, reactants or catalysts might experience phase changes, such as transitioning from solid to liquid, potentially altering the reaction pathway. (4) Impact of environmental conditions: Within the constraints of a constant-volume sealed system, the exchange of heat and matter with the external environment is restricted. This limitation may induce alterations in the microenvironment of the reactants, including fluctuations in local temperatures and pressures, each of which could potentially influence ignition energy required.

**Table 4:** Detail table of combustion index of the nanothermites

Nanothermite	Ignition delay/s	Ignition energy/J	Combustion time/s	Peak pressure /MPa	Average pressure rise rate/MPa·s^− 1^
Al/MoO_3_	0.13	2.004	0.03	0.188	6.27
Al/MoO_3_/Kp	0.10	1.505	0.03	0.751	25.03

**Fig. 6:**
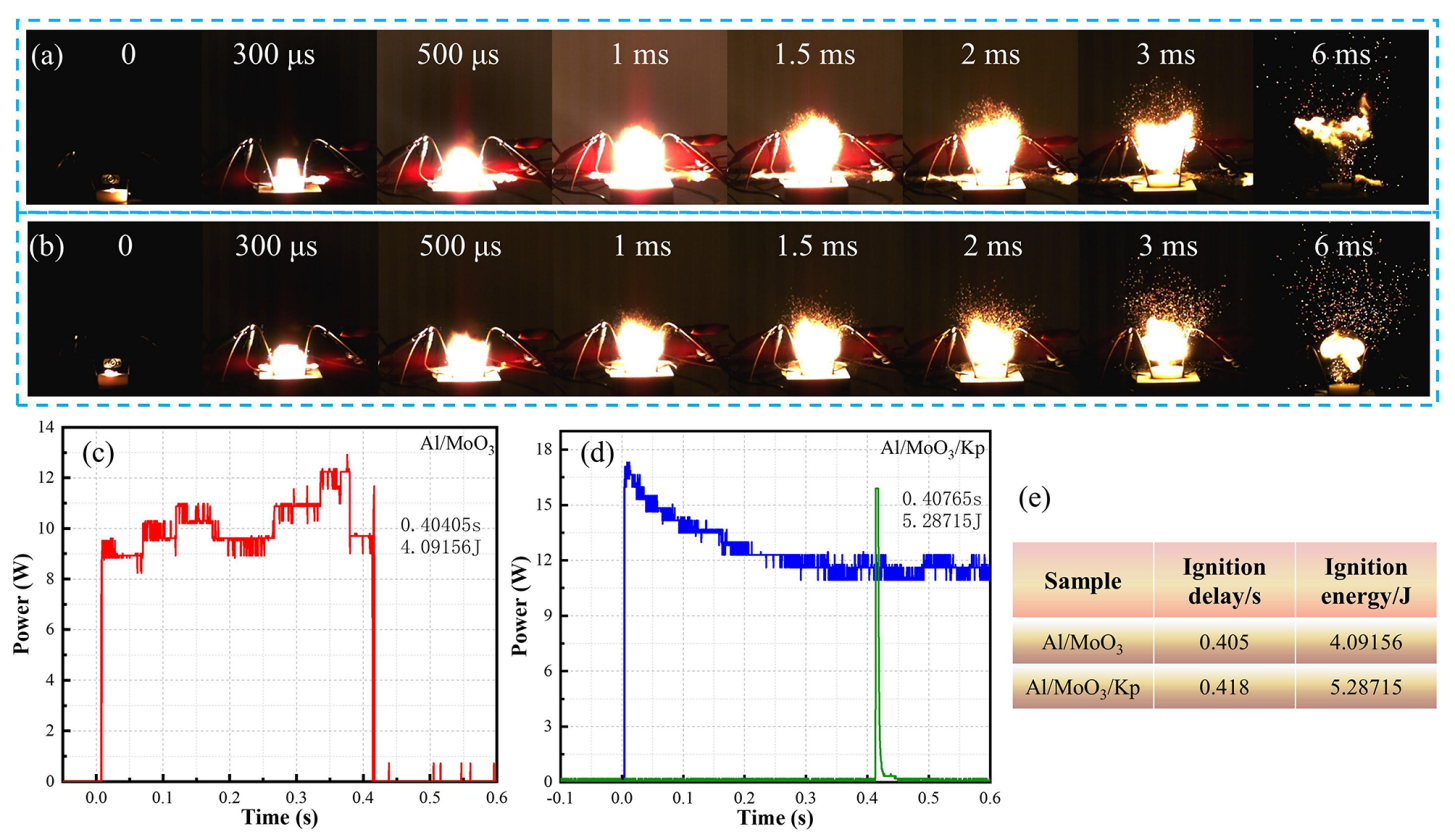
Nanothermite combustion in an open environment (**a**) High speed photography of Al/MoO_3_ combustion (**b**) High speed photography of Al/MoO_3_/Kp combustion (**c**) Power-time characteristic curve of the Al/MoO_3_ nanothermite in the ignition delay stage (**d**) Power-time characteristic curve of the Al/MoO_3_/Kp nanothermite in the ignition delay stage (**e**) Detail table of ignition of nanothermites

To acquire a clearer combustion morphology of the nanothermites, the closed bomb was removed and the combustion process was captured using high-speed photography. The test was a constant pressure combustion one because it was performed in an open environment. The combustion morphology captured by high-speed photography is shown in Fig. 6(a)?∼(b), with the recording time of the initial combustion image as the starting point, which is recorded as time 0.

The two thermites had similar combustion morphology and reaction time, and both reached the most intense moment of combustion at approximately 1 ms, accompanied by bright light and explosion sound. Since the constant pressure combustion took place in an open environment and was not subjected to any constraints, it went rapidly. The thermites almost burned out within 6 ms. However, their combustion processes were partially different.

Unlike the Al/MoO_3_ nanothermite, the combustion of Al/MoO_3_/Kp nanothermite was accompanied by sparks spattering, proving that a large amount of gas was produced during the process, which was also confirmed in the constant volume combustion test. Moreover, The ignition delay time and energy of the Al/MoO_3_ and Al/MoO_3_/Kp nanothermites were slightly different, as shown in Fig. 6(c)?∼(e). The ignition delay time of Al/MoO_3_/Kp nanothermite is slightly longer, and its ignition energy is higher than that of the Al/MoO_3_ nanothermite. Therefore, the combustion of Al/MoO_3_/Kp nanothermite requires more energy and has a certain stability. The results are consistent with the activation energy analysis.

### Characterization of combustion products

**Fig. 7:**
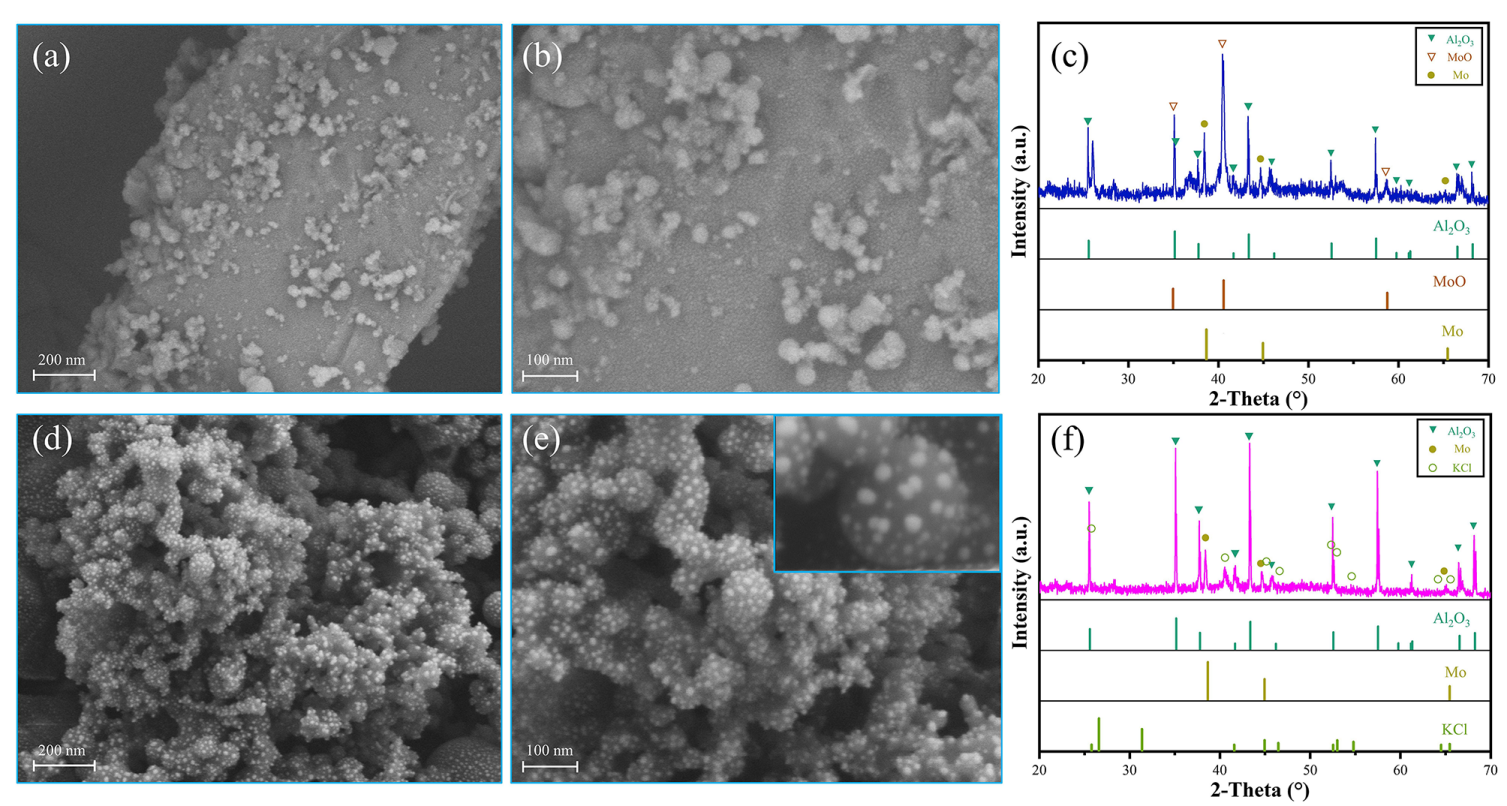
Combustion product characterization diagrams of the nanothermites (**a**) SEM image of Al/MoO_3_ combustion products (**b**) Locally enlarged SEM image of Al/MoO_3_ combustion products (**c**) XRD patterns of Al/MoO_3_ combustion products (**d**) SEM image of Al/MoO_3_/Kp combustion products (**e**) Locally enlarged SEM image of Al/MoO_3_/Kp combustion products (f) XRD patterns of Al/MoO_3_/Kp combustion products

Figure 7 exhibit the SEM electron micrographs and XRD images of the combustion products of the nanothermites. Figure 7(a)?∼(b) show the microscopic SEM images of the combustion products of Al/MoO_3_ nanothermite, exhibiting small particles uniformly adhered to the nano-rod. XRD detection, as shown in Fig. 7(c), reveals that the products comprise Al_2_O_3_ (JCPDS No.74-1081), MoO (JCPDS No.78–0424), and Mo (JCPDS No.88-2331). This result indicates that the aluminothermic reaction occurred during the combustion process of Al/MoO_3_ nanothermite, whereas MoO_3_ was not completely reduced, and this reaction was not complete. Based on the SEM image, it could be speculated that the rod was the produced Mo and the incompletely reduced MoO.

The combustion products of Al/MoO_3_/Kp nanothermite resemble a dense “coral”, as shown in Fig. 7(d)?∼(e). This is possibly the effect of reactants melt and then solidification during the combustion process. The enlarged view of a sphere in Fig. 7(e) shows many white dots adhering to its surface. These white dots evenly cover the entire “coral-like” surface. Figure 7(f) is the XRD pattern of the combustion products of Al/MoO_3_/Kp, which comprise Al_2_O_3_, Mo, and KCl. Previous studies have found that the melting and thermal decomposition temperature for KClO_4_ is approximately 570^o^C [30, 43, 44], at which it decomposes into KCl and O_2_. The generation of a large amount of O_2_ gas is consistent with the results in the constant volume tests. The melting point of KCl is approximately 770^o^C [20, 45]. During the combustion process, the temperature is high enough to reach this melting point. Therefore, the “coral-like” product forms by solidifying after melting. From the obtained experimental results, it can be deduced that the observed molten “coral-like” formations are composed of KCl. The produced Mo appears to have undergone melt blending with the KCl. The white dot-like substances observed on the outer surface have been identified as Al_2_O_3_.

## Discussion

**Fig. 8:**
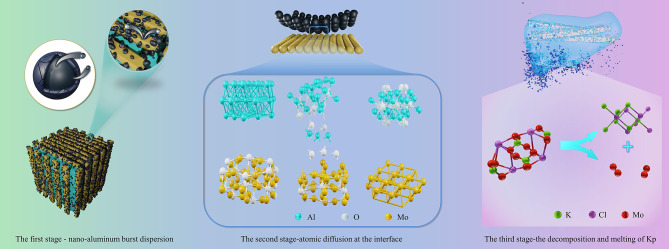
Combustion mechanism diagram of the Al/MoO_3_/Kp nanothermite

The excellent thermal and combustion performance of Al/MoO_3_/Kp nanothermite is closely related to its distinctive reaction mechanism. During the rapid heating process, Al/MoO_3_/Kp nanothermite mainly undergoes three stages of reaction, and its main reaction mechanism is shown in Fig. 8.

The first stage is the burst diffusion stage of nano Al from the oxide layer. In the published literature, there are three prevailing views on the diffusion mechanism of nano Al covered with an oxide layer: diffusion-oxidation mechanism [46, 47], ion diffusion mechanism [48], and melting-diffusion mechanism [49, 50].

The diffusion-oxidation mechanism is that when the heating rate is low, the pressure gradient affects the diffusion of the central Al, i.e., when nano Al is oxidized, the Al atoms in the nucleus diffuse outward and the oxygen atoms diffuse inward [51, 52]. The ion diffusion mechanism suggests that the ignition of Al nanoparticles covered with an oxide layer is mainly due to the internal electric field generated by the alumina layer on the surface, which accelerates the diffusion rate of Al ions through the oxide layer and enhances their diffusion strength. This Intrinsic electric field drives approximately 90% of Al ion mass through [48, 53]. The melting-diffusion mechanism refers to the phenomenon where at extremely high heating rates, the volume of the nucleus expands, the pressure increases, the oxide layer splits, and the liquid-phase aluminum disperses into small liquid-phase clusters that fly off at high speeds [54–56]. The combustion test was realized by rapid heating with an electric current, so the ion diffusion mechanism and melting-diffusion mechanism might occur. According to the SEM images of the combustion products in Fig. 7(a)?∼(b), it can be inferred that nano Al droplets must have splashed during the combustion process. Therefore, the melting-diffusion mechanism was more likely to occur.

The combustion of nanothermite was rapidly heated by electric current to stimulate the reaction. In the process of continuous conversion of electric energy into thermal energy, the temperature in the thermite increased continuously, which made the nano Al covered with an alumina protective layer burst and disperse out of the layer. The nano Al droplets dropped on the reactants, allowing the nano Al to come into close contact with the reactants, as shown in the first stage of Fig. 8.

The second stage is the atomic diffusion behavior at the interface between nano Al and the oxidant, i.e., the rapid redox reaction dominated by the metal-oxygen flipping mechanism at the interface [55]. In this stage, the Al atoms in nano Al and O atoms in MoO_3_ diffused with each other and rapidly combined to form Al-O bonds [57]. Since the reaction occurred at the interface, nano Al was extremely close to the reactants, resulting in a fast reaction rate, as shown in the second stage of Fig. 8.

The third stage is the most important. The published studies have all concluded that this stage is a slow reaction dominated by atomic diffusion motion, which is mainly due to the long mass transfer distance, thus resulting in low reaction efficiency. In the Al/MoO_3_/Kp nanothermite, the rapid melt and decomposition of Kp enables the reaction to proceed efficiently.

It is well known that increasing the contact area between the reactants or the pressure can significantly improve reaction efficiency. A large number of studies have shown that the melting and low-temperature thermal decomposition temperature of KClO_4_ is about 570 ^o^C [30, 43, 44], where it changes frome solid to liquid and decomposes into KCl and O_2_. The peak of the aluminothermic reaction at the interface is about 576 ^o^C (Fig. 4 (b)). At this time, Kp rapidly melts and decomposes into KCl and O_2_. On the one hand, Kp melted into liquid and wrapped Al and MoO_3_ tightly, making the contact between the reactants closer and accelerating the reaction; on the other hand, Kp continuously decomposed into O_2_, leading to an increase in local pressure, which rapidly increased the pressure in the constant volume combustion test (Fig. 5(b)). Simultaneously, splashing sparks could be seen in the combustion morphology (Fig. 6(b)). Moreover, O_2_ provided an oxidizing agent, which promoted the reaction, increased the reaction efficiency, greatly improved the reaction rate of the third stage, and even converted the third stage into the reaction at the interface, which greatly improved the efficiency of the aluminothermic reaction. It could be inferred from the combustion products that all reactants were completely reacted, demonstrating excellent combustion performance. When the reaction was completed, the reactants exhibited a unique morphology of “coral-like” (Fig. 7(e)).

In the case of Al/MoO_3_ nanothermite, after the nano Al burst and dispersed out of the protective layer in the first stage, the second stage of the reaction occurred at the interface between Al and MoO_3_, however, in the third stage, the reaction did not fully proceed due to discontinuous reaction medium or excessively sparse nano Al droplets, resulting in an incomplete reduction of MoO_3_ and the presence of MoO in the combustion products.

In the DSC test, nano Al tended to diffuse from the protective layer through the diffusion-oxidation mechanism due to the slow heating. Afterwards, similar to the combustion reaction, the melt and decomposition of Kp increased the contact area between the reactants. O_2_ was produced to provide an oxidant, which increased the internal pressure of the reactants and promoted the progress of the reaction, facilitating the complete reaction between reactants. Therefore, a large amount of heat was released, demonstrating good thermal performance.

## Conclusions

In this study, the Al/MoO_3_/Kp and Al/MoO_3_ nanothermites were prepared using the ultrasonic dispersion method. Then the prepared nanothermites were tested for their thermal and combustion properties. Thermomechanical analysis was conducted, and the reaction mechanism of Al/MoO_3_/Kp nanothermite was investigated.

The three main stages of the Al/MoO_3_/Kp nanothermite reaction were the bursting reaction where nano Al underwent melt and diffusion, the atomic diffusion at the interface between the reactants, and the rapid melt and decomposition of Kp. The distinctive third stage was the key to excellent thermal and combustion properties. The reactants were melted and wrapped and O_2_ was produced to increase pressure, thus the aluminothermic reaction was promoted comprehensively, leading to a heat release of 1976 J·g^− 1^, a combustion peak pressure of 0.751 MPa, and an extremely fast average pressure rise rate of 25.03 MPa·s^− 1^. Its performance is much better than Al/MoO_3_ nanothermite, the control group. Moreover, the Ea of Al/MoO_3_/Kp nanothermite is 269.66 kJ·mol^− 1^, which is more stable and safer than the 205.64 kJ·mol^− 1^ of Al/MoO_3_ nanothermite. The strategy of altering the physical states of reactants during the reaction process, though demanding precise manipulation and high-accuracy equipment, offers crucial insights for the development of novel, superior performance nanothermites. This innovative approach is informative and holds significant potential for the advancement of nanothermite performance.

## Data Availability

All data generated or analyzed during this study are included in this article. The data that support the findings of this study are available from the corresponding author, [author initials], upon reasonable request.
